# Spinal Cystic Echinococcosis

**DOI:** 10.4269/ajtmh.18-0588

**Published:** 2019-01

**Authors:** Brett S. Mansfield, Kim Pieton, Sugeshnee Pather

**Affiliations:** 1 Department of Internal Medicine, Chris Hani Baragwanath Academic Hospital, Johannesburg, South Africa;; 2 Department of Internal Medicine, Faculty of Health Sciences, University of the Witwatersrand, Johannesburg, South Africa;; 3 Division of Anatomical Pathology, National Health Laboratory Services, Chris Hani Baragwanath Academic Hospital, Johannesburg, South Africa

A 38-year-old South African male, with no prior medical or surgical history, presented with chronic lower back pain which had been present for the past 14 months. The pain was initially intermittent but had worsened and began radiating down the back of his legs. He had no weakness in his legs and was continent of both bladder and bowel. Physical examination was unremarkable apart from slightly brisk lower limb reflexes. He had normal power, and sensation was intact.

**Figure 1.**
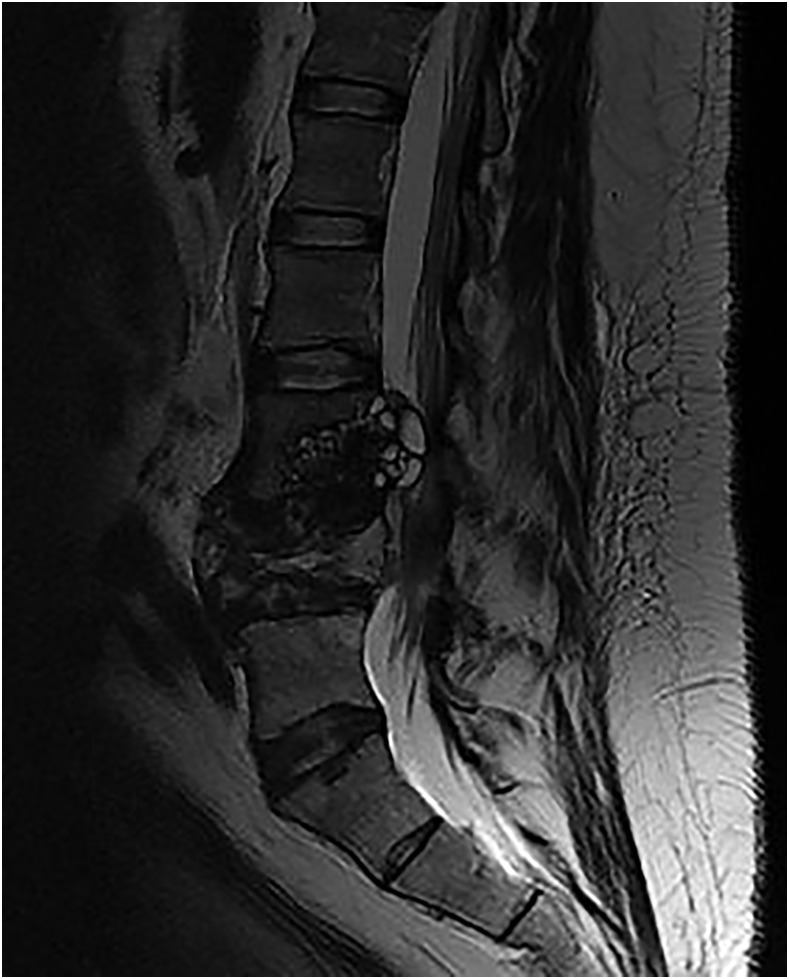
Sagittal magnetic resonance imaging (T2-weighted) of the lumbosacral spine showing an expansile multicystic lesion causing destruction of the fourth lumbar vertebra.

**Figure 2.**
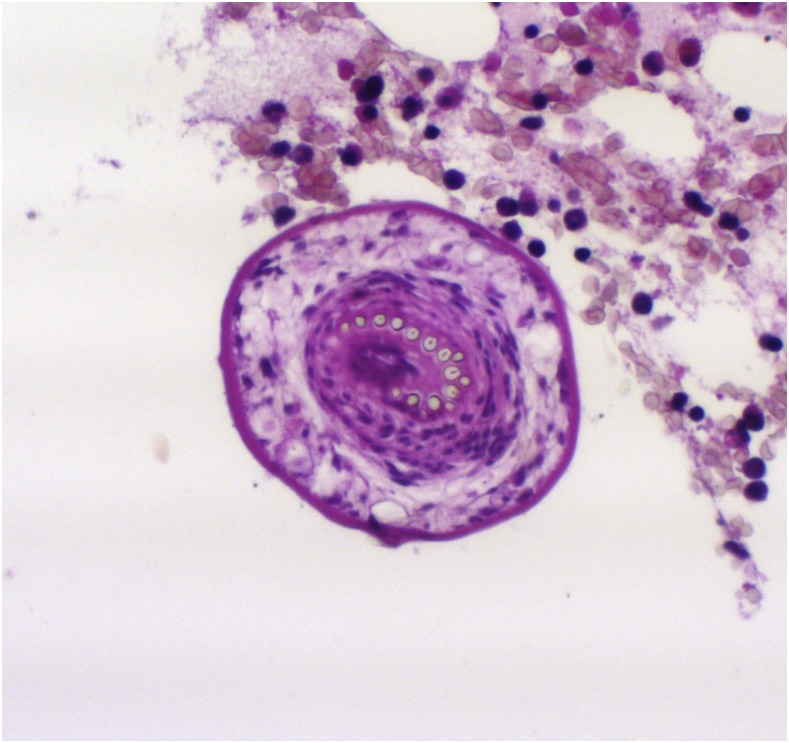
Microscopic image of a protoscolex (the future tapeworm head) amidst bone marrow elements (hematoxylin and eosin stain, ×200 magnification). This figure appears in color at www.ajtmh.org.

Laboratory investigations revealed a mildly elevated white cell count (11.05 × 10^9^/L). Erythrocyte sedimentation rate (3 mm/hour) and C-reactive protein (9 mg/L) were not raised. Magnetic resonance imaging of the spine (Figure 1) revealed vertebral body destruction of the fourth lumbar vertebra with multiple surrounding cysts. Histopathological examination (Figure 2) confirmed the presence of parasitic structures amidst lamellar bone and marrow elements. There were parasitic cysts comprising a laminated periodic acid-Schiff-positive wall, daughter cysts, and protoscolices. Hooklets within the protoscolices were refractile and acid-fast characteristics were highlighted by the modified Ziehl-Neelsen stain. These features were supportive of hydatid cysts.

The patient was started on albendazole 400 mg orally twice daily and underwent a vertebrectomy with insertion of a vertebral cage. He was discharged home a few weeks later.

Cystic echinococcosis (CE), or hydatid disease, most commonly affects the liver (70%) and lungs (20%).^1^ Spinal CE occurs in less than 1% of all cases.^2^ The disease may remain asymptomatic for many years and only become evident following a pathological fracture or neurological deficit.

Management entails surgical excision and at least 6 months of albendazole therapy. Only 30–40% of patients with spinal CE make a full recovery and the disease has high rates of morbidity and mortality.^3^ Recurrence rates are high, with 48% of those with vertebral disease having evidence of disease recurrence at 24 months.^3^ It is for this reason that close follow-up with serial imaging is required.
